# Supplementary material for: A Late Cretaceous amber biota from central Myanmar
Source: Nat Commun. 2018 Aug 9;9:3170. doi: 10.1038/s41467-018-05650-2 (PMC6085374; doi:10.1038/s41467-018-05650-2)
Supplement: Supplementary file 1 — Supplementary Information [file 41467_2018_5650_MOESM1_ESM.pdf]

Supplementary Information

A Late Cretaceous amber biota from central Myanmar

Zheng et al.

Supplementary Note 1

Both Tilin and Kachin ambers were closely located in West Burma block, which was near the equator during Late Cretaceous (Supplementary Figure 1). Regionally, Tilin amber mining is in Tilin (21° 41' N, 94° 5' E), Gangaw district, Magway region of central Myanmar (Supplementary Figure 2a), while Kachin amber mines are distributed in the Hukawng Valley of northern Myanmar (Supplementary Figure 2b). In Tilin, more than 30 pits have been made along the hill baron, which cover an area about 10 km^2 (Supplementary Figure 3; also see Tay et al.[1]). A typical pit is generally 1 m wide and 10–20 m deep to reach amber-bearing layers (Supplementary Figure 3b). The amber samples were mined from the Cretaceous Kabaw Formation, and are preserved in coal seams beneath a grey tuff and above a yellow sandstone intercalated by conglomerates near mine surface (Supplementary Figure 4).

The present paper reports on the study of 5 kg of raw amber pieces that were collected from a single coal seam. The samples were ground and polished manually using a series of wet silicon carbide papers to produce smooth clean surfaces for investigation of fossil inclusions. One sediment sample (M-1) weighing about 5 kg was collected from the tuff just overlying or somewhat interbedded with the amber-bearing coal seam, and processed for LA-(MC)-ICP-MS U-Pb dating. Three ammonites (*Sphenodiscus* sp.) preserved in concretions within the sandstone were also collected. *Sphenodiscus* sp. has the whorl section stouter than that of *S. lotatus*, and the external suture with the adventive lobes much smaller than the lateral so that the adventive saddles are rather small and has lesser incisions than *S. lotatus*. *Sphenodiscus* sp. resembles *S. ubaghsi*, but differs in having obviously ventrolateral tubercles. Several amber fragments were processed for Pyrolysis Gas Chromatography Mass Spectrometry analysis.

Significant insect faunas known from the Canadian amber of the Campanian Foremost Formation, occurred approximately 13 million years before the KPg boundary[2]. The Fur Formation, a Danish unit that contains insect compression fossils from approximately 11 million years after the KPg event[3]. There are few amber sites recorded from the upper Campannian to Maastrichtian. Until now, it has been reported only six times, from the Danek Bonebed in Canada, the Modena region in Italy, the Corbières region in France, the Tremp Formation in Spain, and the Fruitland Formation and Hell Creek Formation in the United States[4–9]. Most of these ambers are devoid of biological inclusions, and few insects have been recognized only in Hell Creek amber (22 specimens, mostly dipterans[8]). Our discovery of fossils in the latest Campanian amber of central Myanmar reveals a diverse insect biota and provides a rare insight into a latest Campanian forest ecosystem.

Supplementary Figures

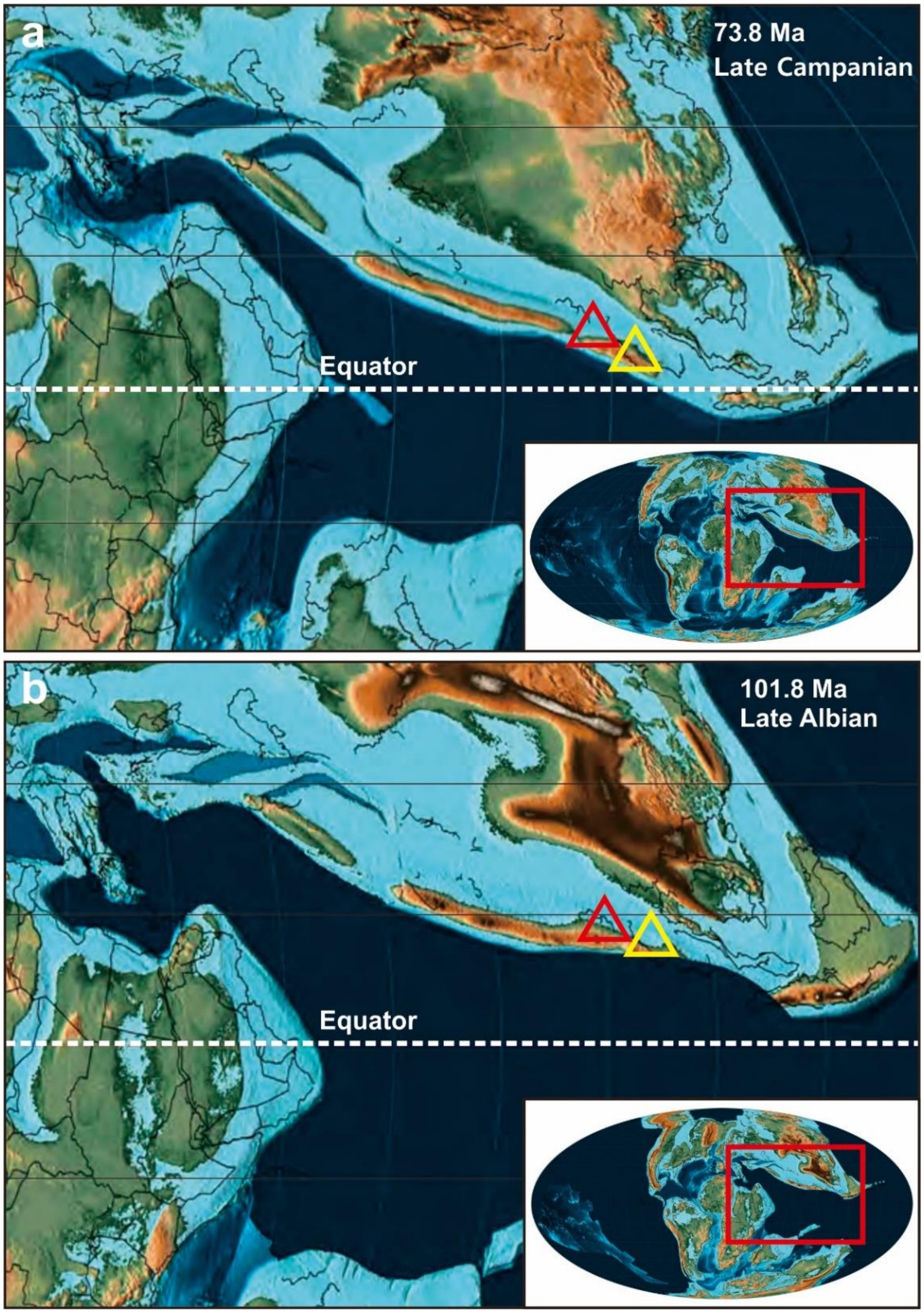

Supplementary Figure 1 | Palaeogeographic positions of Tilin and Kachin amber sites. a, palaeogeographic map during late Campanian; **b**, palaeogeographic map during late Albian (based on Scotese[10]). Yellow triangle, Tilin amber site; red triangle, Kachin amber site.

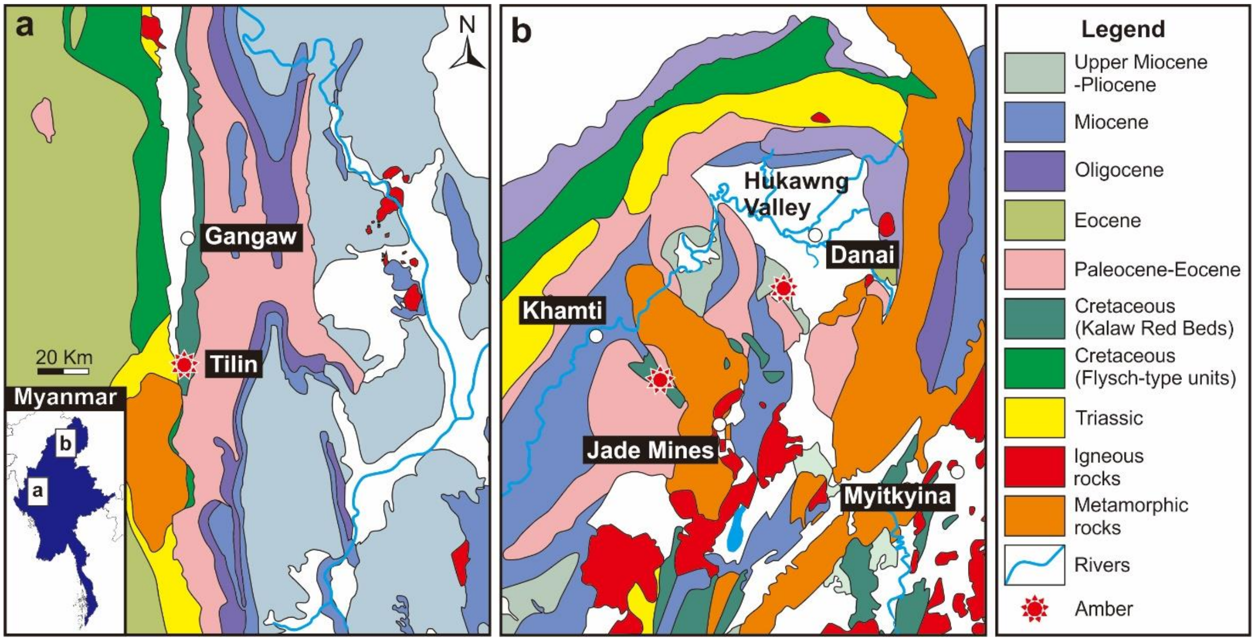

Supplementary Figure 2 | Geological maps showing amber positions in Myanmar. a, Geological location of Tilin amber in Gangaw, Magway of central Myanmar; **b**, Geological location of Kachin amber in Hukawng Valley, northern Myanmar.

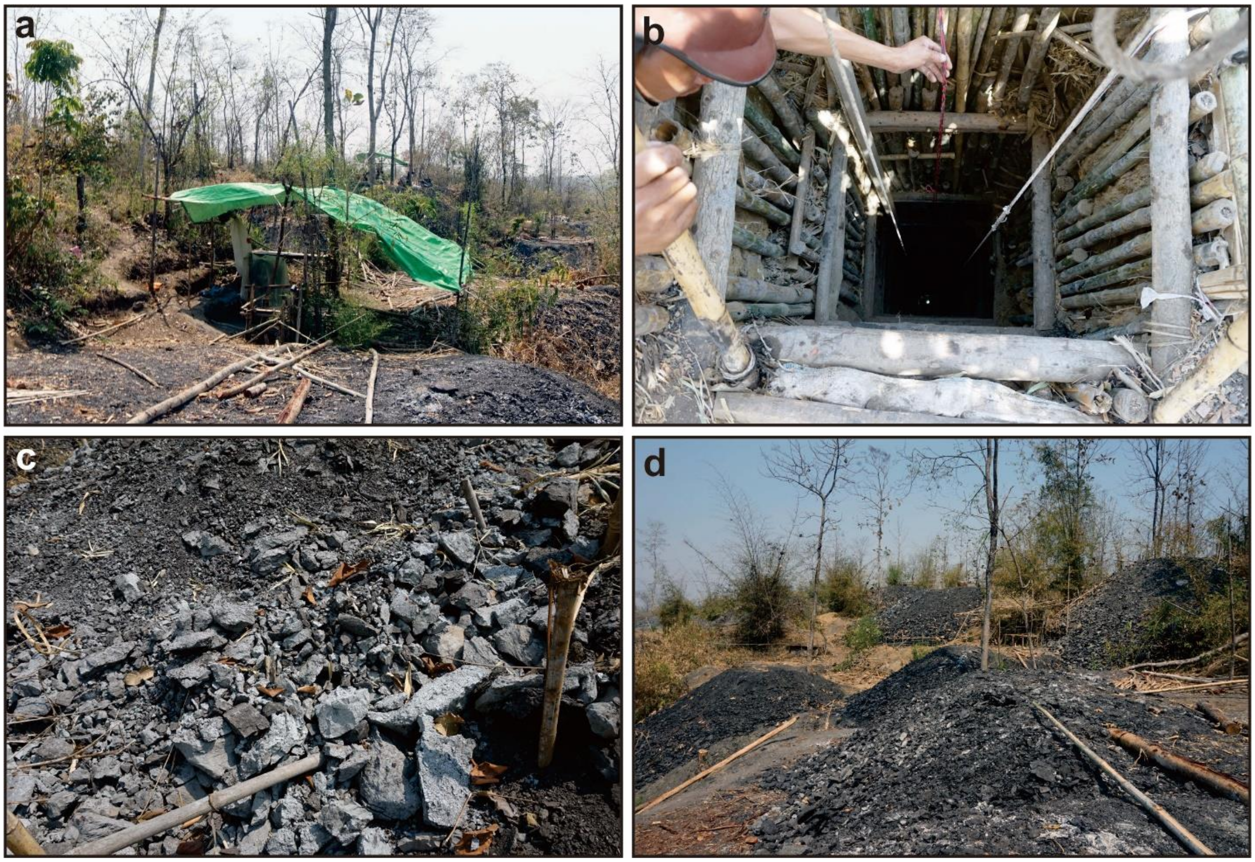

Supplementary Figure 3 | Photograph showing outcrops of Tilin amber site. a, distribution of amber mines along the hill; **b**, top view of an amber mine; **c**, tuff excavated from amber mines; **d**, coal seam excavated from amber mines.

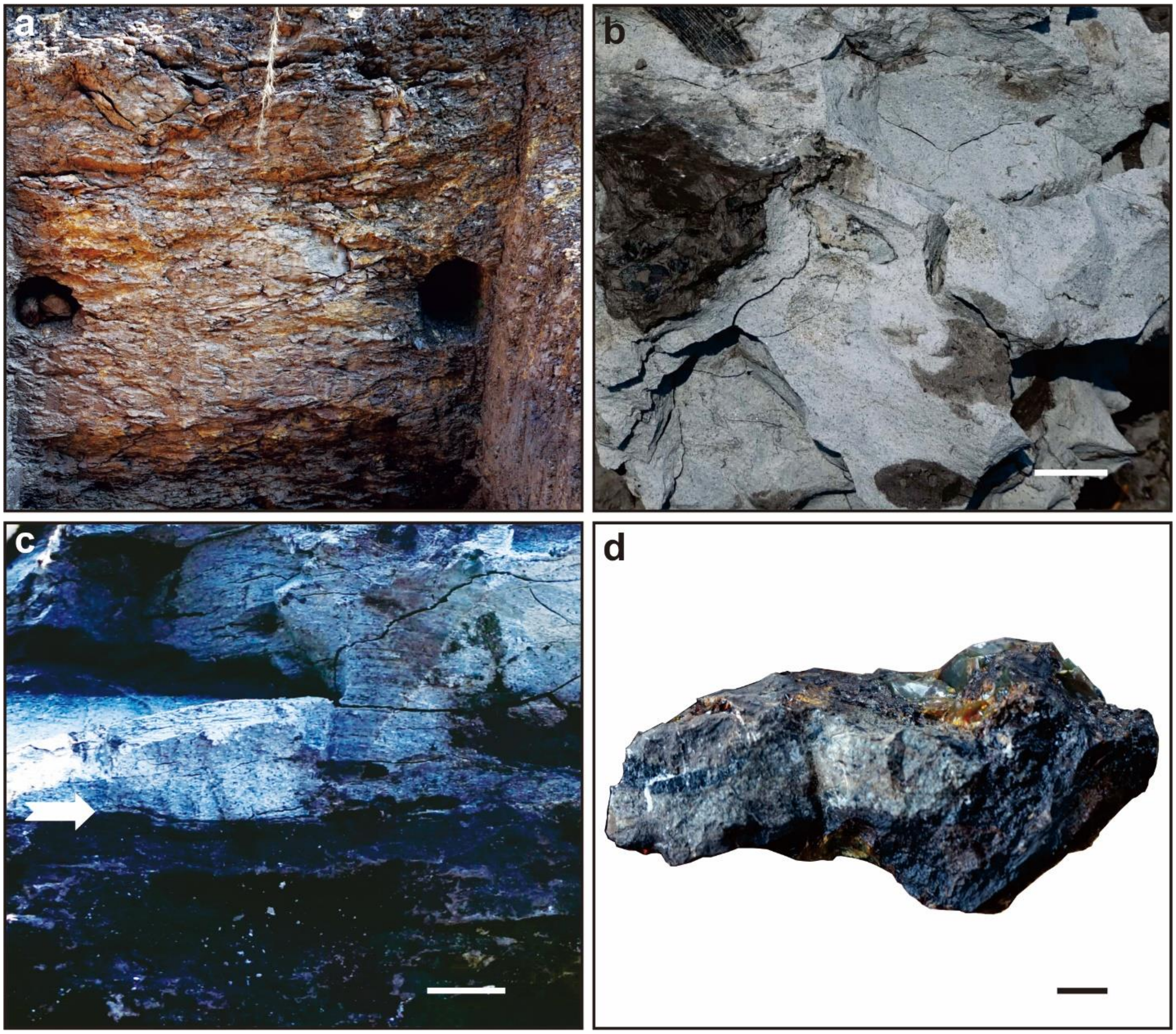

Supplementary Figure 4 | Photographs showing lithology of Tilin amber site. a, soil near mine surface; **b**, tuff bearing some charcoal; **c**, boundary between tuff and coal seam (marked by white arrow); **d**, raw amber as found in coal seam. Scale bars, 10 mm.

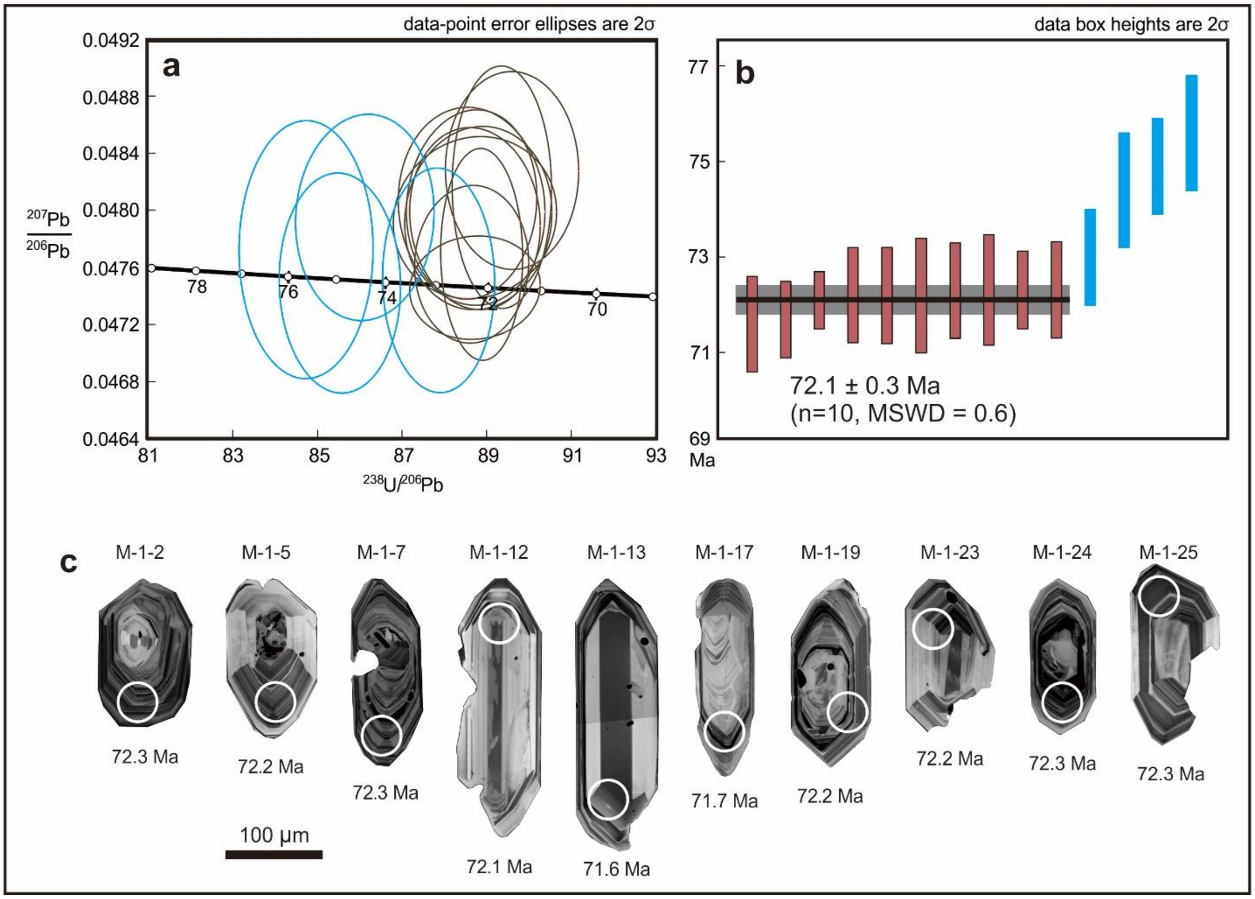

Supplementary Figure 5 | U-Pb geochronology of M-1 from Tilin amber. a, Tera-Wasserburg plot for zircons from M-1; **b**, rank order plot for zircons from M-1 showing weighted mean calculated ^{206}Pb/^{238}U dates; **c**, cathodoluminescent (CL) images of ten young zircons analyzed in **b** (red circle diameter= 40 μm). MSWD—mean square of weighted deviates.

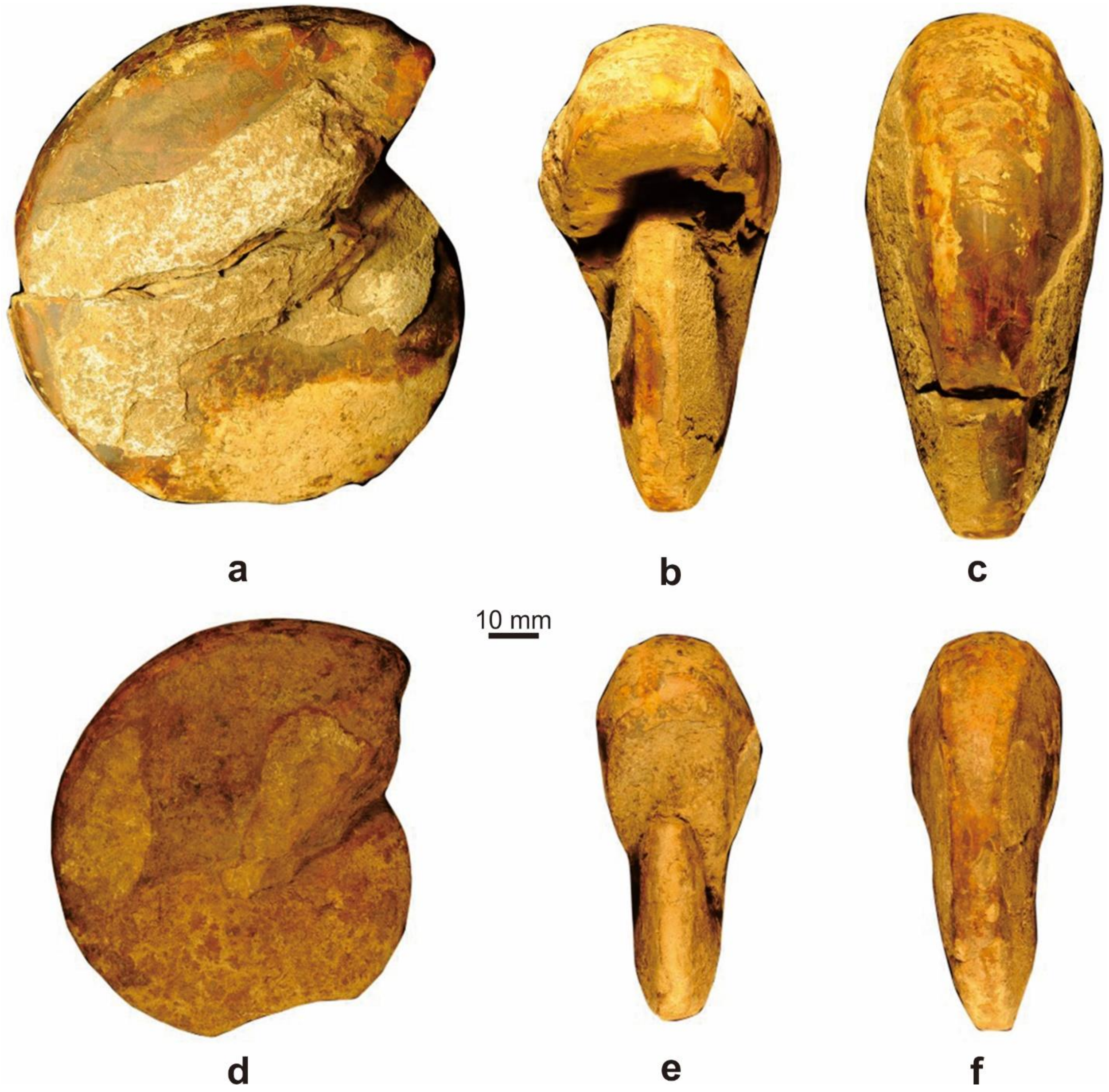

Supplementary Figure 6 | Photographs of *Sphenodiscus* sp. a–c, specimen A, NIGP168515; **a**, lateral view; **b**, front view; **c**, ventral view; **d–f**, specimen B, NIGP168516; **d**, lateral view; **e**, front view; **f**, ventral view.

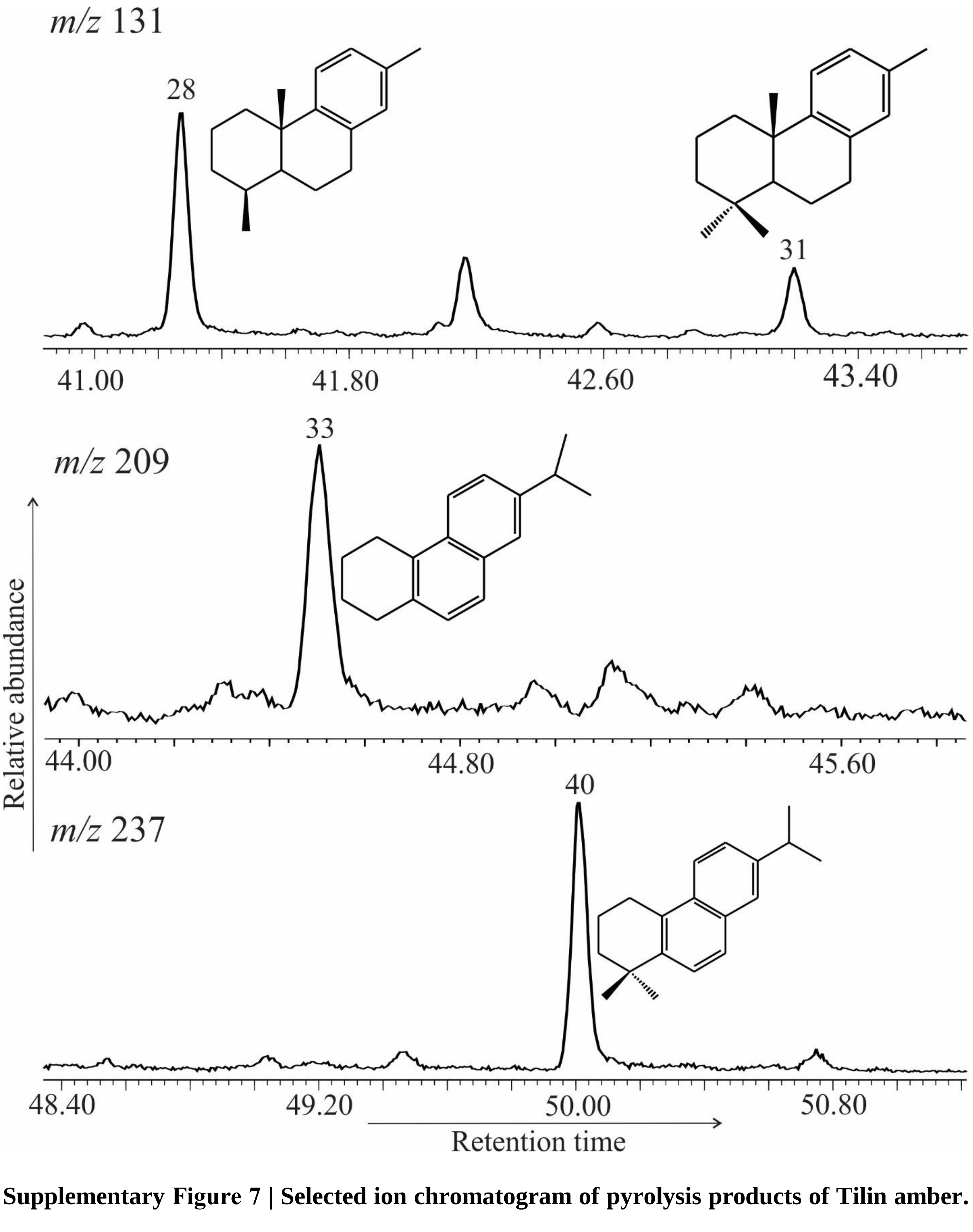

Supplementary Figure 7 | Selected ion chromatogram of pyrolysis products of Tilin amber. Selected number peaks: 28 (16, 17, 18-trisnorabieta-8, 11, 13-triene), 31 (bisnordehydroabeitane), 33 (bisnorsimonellite), and 40 (simonellite).

Supplementary Table 1 | U-Pb analytical results for tuff sample from Hti Lin amber mine.

Samples	Isotopic ratios							rho	U-Pb Ages(Ma)						concor.
	Th/U	$^{207}Pb/^{206}Pb$	±1σ	$^{207}Pb/^{235}U$	±1σ	$^{206}Pb/^{238}U$	±1σ		$^{207}Pb/^{206}Pb$	±1σ	$^{207}Pb/^{235}U$	±1σ	$^{206}Pb/^{238}U$	±1σ	
**M-1, tuff overlying amber-bearing layers**															
91500	0.49	0.075	0.0003	1.850	0.0109	0.179	0.0008	0.797	1070	5	1063	4	1060	5	99%
91500	0.50	0.075	0.0003	1.851	0.0101	0.180	0.0008	0.768	1061	5	1064	4	1065	4	99%
91500	0.50	0.075	0.0003	1.850	0.0112	0.180	0.0008	0.753	1056	6	1063	4	1067	4	99%
91500	0.49	0.075	0.0004	1.850	0.0112	0.178	0.0007	0.672	1074	6	1064	4	1058	4	99%
91500	0.50	0.075	0.0003	1.850	0.0108	0.179	0.0009	0.822	1070	5	1063	4	1060	5	99%
91500	0.49	0.075	0.0003	1.851	0.0116	0.180	0.0009	0.765	1060	6	1064	4	1065	5	99%
91500	0.45	0.075	0.0003	1.852	0.0110	0.179	0.0009	0.805	1065	5	1064	4	1064	5	99%
91500	0.49	0.075	0.0003	1.850	0.0117	0.179	0.0009	0.808	1063	6	1063	4	1063	5	99%
91500	0.49	0.075	0.0003	1.849	0.0101	0.179	0.0009	0.876	1068	5	1063	4	1061	5	99%
GJ-1	0.05	0.060	0.0003	0.815	0.0067	0.098	0.0007	0.874	606	8	605	4	604	4	99%
GJ-1	0.05	0.060	0.0004	0.813	0.0097	0.098	0.0010	0.857	611	12	604	5	602	6	99%
GJ-1	0.05	0.060	0.0002	0.817	0.0050	0.099	0.0005	0.808	596	6	606	3	609	3	99%
GJ-1	0.05	0.060	0.0002	0.816	0.0051	0.098	0.0006	0.932	609	6	606	3	605	3	99%
GJ-1	0.05	0.060	0.0002	0.815	0.0050	0.098	0.0005	0.818	608	6	605	3	605	3	99%
GJ-1	0.05	0.060	0.0002	0.816	0.0052	0.098	0.0005	0.853	618	6	606	3	602	3	99%
GJ-1	0.05	0.060	0.0002	0.817	0.0050	0.099	0.0006	0.944	604	6	607	3	607	3	99%
GJ-1	0.05	0.060	0.0002	0.818	0.0051	0.099	0.0005	0.887	608	6	607	3	607	3	99%
M-1-1	0.75	0.048	0.0005	0.078	0.0008	0.012	0.0001	0.701	89	13	75.9	0.8	75.6	0.6	99%
M-1-2	0.72	0.048	0.0003	0.075	0.0007	0.011	0.0001	0.764	101	10	73.2	0.6	72.3	0.5	98%
M-1-3	0.87	0.083	0.0367	0.127	0.0559	0.011	0.0006	0.490	1260	1005	121	50	71	4	-40%
M-1-4	0.75	0.049	0.0004	0.076	0.0007	0.011	0.0001	0.663	144	11	74.4	0.7	72.2	0.4	97%
M-1-5	1.14	0.048	0.0003	0.074	0.0007	0.011	0.0001	0.815	98	10	72.9	0.7	72.2	0.5	99%
M-1-6	0.72	0.047	0.0003	0.077	0.0007	0.012	0.0001	0.730	73	11	74.9	0.7	74.9	0.5	99%
M-1-7	0.78	0.048	0.0003	0.074	0.0006	0.011	0.0001	0.789	82	8	72.5	0.5	72.3	0.4	99%
M-1-8	0.76	0.049	0.0012	0.075	0.0018	0.011	0.0001	0.544	128	60	73	2	71.4	0.5	92%
M-1-9	0.78	0.048	0.0003	0.077	0.0007	0.012	0.0001	0.772	95	11	75.1	0.7	74.4	0.6	99%

M-1-10	0.70	0.049	0.0005	0.078	0.0009	0.012	0.0001	0.612	132	15	75.8	0.9	74	0.5	97%
M-1-11	0.68	0.049	0.0006	0.077	0.0012	0.011	0.0001	0.673	135	19	75	1	73.5	0.8	97%
M-1-12	0.80	0.048	0.0003	0.074	0.0006	0.011	0.0001	0.588	84	10	72.5	0.6	72.1	0.3	99%
M-1-13	0.62	0.048	0.0004	0.074	0.0007	0.011	0.0001	0.777	113	10	72.8	0.6	71.6	0.5	98%
M-1-14	0.37	0.058	0.0002	0.671	0.0062	0.084	0.0008	1.007	520	9	521	4	522	5	99%
M-1-15	0.81	0.050	0.0003	0.077	0.0010	0.011	0.0001	0.720	185	14	75.7	0.9	72.1	0.6	95%
M-1-16	0.92	0.051	0.0013	0.076	0.0019	0.011	0.0001	0.543	229	62	75	2	69.8	0.5	81%
M-1-17	0.66	0.048	0.0003	0.074	0.0007	0.011	0.0001	0.604	106	11	72.8	0.6	71.7	0.4	98%
M-1-18	0.68	0.050	0.0004	0.079	0.0009	0.012	0.0001	0.688	177	12	77.3	0.8	74.1	0.5	95%
M-1-19	0.75	0.047	0.0002	0.074	0.0006	0.011	0.0001	0.912	73	8	72.2	0.5	72.2	0.5	99%
M-1-20	0.67	0.048	0.0004	0.075	0.0007	0.011	0.0001	0.685	75	11	73	0.7	73	0.5	99%
M-1-21	0.67	0.049	0.0004	0.077	0.0009	0.011	0.0001	0.673	127	14	75.1	0.9	73.5	0.6	97%
M-1-22	0.81	0.053	0.0072	0.081	0.0108	0.011	0.0002	0.202	348	303	79	10	71	1	44%
M-1-23	0.59	0.048	0.0003	0.074	0.0007	0.011	0.0001	0.812	96	10	73	0.7	72.3	0.5	99%
M-1-24	0.80	0.048	0.0002	0.075	0.0007	0.011	0.0001	0.871	96	10	73	0.7	72.2	0.6	99%
M-1-25	0.64	0.048	0.0003	0.075	0.0008	0.011	0.0001	0.816	99	11	73.1	0.7	72.3	0.6	98%

Supplementary Table 2 | Major compounds identified from the Py-GC-MS analysis of Tilin amber.

1	Benzene	21	C_4 Naphthalene
2	Toulene	22	C_4 Naphthalene
3	C_2 benzene	23	Rearranged C_{19} tricyclic terpane
4	C_2 benzene	24	Rearranged C_{19} tricyclic terpane
5	C_3 benzene	25	C_{19} tricyclic terpane
6	C_3 benzene	26	Phenanthrene
7	Dihydronaphthalene	27	C_{19} tricyclic terpane
8	Naphthalene	28	16,17,19 -Trisnorabieta-8,11,13-triene
9	Benzene, (3-methyl-2-butenyl	29	Regular C_{19} tricyclic terpane
10	Naphthalene, 1,2-dihydro-3-methyl-	30	Methyl Phenanthrene
11	C_1 Naphthalene	31	Bisnordehydroabeitane
12	C_1 Naphthalene	32	Methyl Phenanthrene
13	Naphthalene, 1,2,3,4-tetrahydro-1,5-dimethyl-	33	Bisnorsimonellite
14	C_2 Naphthalene	34	*n*-C20
15	C_2 Naphthalene	35	Ethyl phenanthrene
16	C_2 Naphthalene	36	1,2,3,4-Tetrahydroretene
17	Ionene	37	C_{16} Sesquiterpane
18	Methyl Ionene	38	*n*-C21
19	Naphthalene, 1,4-dihydro-2,5,8-trimethyl-	39	Trimethylphenanthrene
20	C_3 Naphthalene	40	Simonellite

Supplementary References

1. Tay, T. S. *et al*. Burmese amber from Hti Lin. *J. Gemmol.* **34**, 606–615 (2015).

2. McKellar, R. C. & Engel, M. S. Hymenoptera in Canadian Cretaceous amber (Insecta). *Cretaceous Res.* **35**, 258–279 (2012).

3. Rust, J. & Andersen, N. M. Giant ants from the Paleogene of Denmark with a discussion of the fossil history and early evolution of ants (Hymenoptera: Formicidae). *Zool. J. Linn. Soc.* **125**, 331–348 (1999).

4. Bellis, D. & Wolberg D. L. Analysis of gaseous inclusions in fossil resin from a Late Cretaceous stratigraphic sequence. *Palaeogeogr. Palaeoclimatol. Palaeoecol.* **97**, 69–82 (1991).

5. Nel, A., De Ploëg, G., Millet, J., Menier, J.-J. & Waller, A. The French ambers: a general conspectus and the Lowermost Eocene amber deposit of Le Quesnoy in the Paris Basin. *Geol. Acta* **2**, 3–8 (2004).

6. Delclòs, X., *et al*. Fossiliferous amber deposits from the Cretaceous (Albian) of Spain. *C. R. Palevol* **6**, 135–149 (2007).

7. DePalma, R., Cichocki, F., Dierick, M. & Feeney, R. Preliminary notes on the first recorded amber insects from the Hell Creek Formation. *J. Paleontol. Sci.* **C.10.0001**, 7 (2010).

8. Davies, L. J., McKellar, R. C., Muehlenbachs, K. & Wolfe, A. P. Isotopic characterization of organic matter from the Danek Bonebed (Edmonton, Alberta, Canada) with special reference to amber. *Can. J. Earth Sci.* **51**, 1017–1022 (2014).

9. Neri, M., *et al*. Nuovi dati stratigrafici sull'ambra di Castelvecchio di Prignano (MO), Paleodays 2016, XVI Giornate di Paleontologia, Faenza, Italy, abstract book p. 63 (2016).

10. Scotese, C. R. *Atlas of Late Cretaceous paleogeographic maps, PALEOMAP atlas for ArcGIS,*

volume 2, The Cretaceous, Maps 16–22, Mollweide Projection (PALEOMAP Project, Evanston, IL, 2014).
